# Mobile cognitive behavioral therapy for insomnia: analysis of factors affecting treatment prognosis

**DOI:** 10.1038/s41598-024-53119-8

**Published:** 2024-02-07

**Authors:** Jia Wei, You Xu, Hongjing Mao

**Affiliations:** Affiliated Mental Health Center & Hangzhou Seventh People’s Hospital, Zhejiang University, Hangzhou, China

**Keywords:** Diseases, Risk factors

## Abstract

This study aims to explore the factors that affect the prognosis of patients with insomnia who are treated with mobile cognitive behavioral therapy. Patients with insomnia who visited the sleep disorders clinic were selected and completed mobile Cognitive behavioral therapy. Patients completed at least three evaluations (including baseline, monthly evaluations thereafter, and a final evaluation at the end of one year) and responded well to treatment within one year of follow-up. Insomnia, anxiety, and depression symptoms were evaluated using the Pittsburgh Sleep Quality Index (PSQI), the Generalized Anxiety Disorder 7-item (GAD-7) scale, and the 9-item Patient Health Questionnaire (PHQ-9), respectively. Treatment prognosis including relapse, recurrence, and remission group. These 339 patients were divided into three groups: 160 patients who remission, 100 patients who relapsed, and 79 patients who experienced recurrence after remission. Demographic characteristics of the 339 patients showed no significant difference in gender between the three groups (*P* = 0.978). However, significant differences were found in age (*P* = 0.006) and onset time (P = 0.000) among the three groups. The remission group had a higher average age than the recurrence group and the relapse group, and the onset time was slower than the other two groups. Multivariate logistic regression analysis showed that age and delayed onset time were protective factors for good treatment prognosis, while low educational level and high GAD-7 scores were independent risk factors for poor prognosis. There are many factors that affect the treatment prognosis of insomnia. Age, low educational level, high GAD-7 scores, and delayed onset time can be used to predict the prognosis of insomnia treatment.

## Introduction

A previous paper has reported that about 25% of adults are dissatisfied with their sleep^1^, 6–15% of people have insomnia symptoms^2^, and 1.6–10% of people meet the diagnostic criteria for insomnia^3^. Chronic insomnia significantly increases the risk of depression, substance abuse, and even suicide. It also increases the risk of medical conditions such as diabetes, hypertension, and myocardial infarction^4,5^. Insomnia consumes a large number of medical resources and reduces quality of life^6^, making it increasingly regarded as a major public health problem worldwide.

Cognitive-behavioral therapy for insomnia (CBT-I), a non-pharmacotherapy method, uses cognitive techniques to enhance circadian rhythms, enhance sleep drive, and relieve insomnia-related anxiety, thereby improving sleep quality^7^. The effectiveness of CBT-I has been well established, making it widely recognized as a first-line treatment for insomnia and the preferred long-term treatment for sleep disorders^8,9^. However, traditional CBT-I is limited by the lack of professionals, high costs, and time-consuming nature, which hinders its development and is associated with poor patient compliance^10^. Mobile CBT-I is an online version that allows patients to receive CBT-I anytime and anywhere through their mobile phones. Some previous have reported that this treatment is not only as effective as traditional CBT-I, but also covers more populations, and allows for better targeting of the factors contributing to insomnia maintenance, leading to improve sleep efficiency, improve mood, and reduce daytime functional impairment caused by insomnia^11,12^.

Based on the above researches, we realize that mobile-based mobile CBT-I has significant potential in the treatment of insomnia. There are many factors that affect the effectiveness of insomnia treatment, but there are few longitudinal studies to determine these factors. This study real-world study aims to explore the factors that affect the treatment prognosis of insomnia patients undergoing mobile CBT-I in combination with pharmacotherapy.

## Materials and methods

### Study subjects

Patients with insomnia who visited the sleep disorders clinic of Seventh People's Hospital of Hangzhou from January 1, 2017 to September 2020 were selected, completed online CBT treatment on the "Good Sleep 365" platform, which is a mobile application for insomnia patients that allows patients to record sleep diaries and conduct evaluations in the application. Specifically, Good Sleep 365 is a non-drug professional sleep APP for insomnia people to conduct self-assessment and rehabilitation training, according to cognitive behavioral therapy management to improve sleep quality, the product will regularly push professional rehabilitation videos, provide doctors with online telephone consultation. The supplier is Hangzhou Zhilan Health Co., Ltd., version: 4.8.0.

### Sample seize, inclusion and exclusion criteria

The determination of the sample size for our study took into consideration various factors, including the research question, statistical power considerations, and feasibility. Initially, a power analysis was conducted to calculate the required sample size to detect a clinically significant difference between the groups. Subsequently, an assessment was made regarding the availability of eligible participants within a reasonable timeframe. To accomplish this, G*power 3.1.9.7 was utilized, employing t-tests with the following configurations: two-tailed test, effect size of 0.5, alpha level of 0.05, and a desired power of 0.80. Based on these calculations, a sample size of 339 was determined to be adequate for this research.

Inclusion criteria: (1) meeting the diagnostic criteria for insomnia in ICD-10; (2) aged between 18 and 65 years old; (3) able to use the "Good Sleep 365" computer application (APP); (4) Follow-up was 1 year with at least 3 assessments (including baseline surveys, monthly surveys thereafter, and surveys up to 1 year). A total of 1395 patients had a Pittsburgh Sleep Index (PSQI) baseline score of > 9 and were followed up at least once to receive online CBT in combination with medication, and 399 patients (PSQI assessed at least twice ≤ 9) responded to treatment within 1 year of follow-up. According to the treatment efficacy of patients within 1 year, they were divided into the following three groups: remission group: from the onset time (in weeks) of continuous PSQI ≤ 9 points (the first week), to the 52nd week, the total PSQI score was ≤ 9 points. Relapse group: from the onset time, the total PSQI score was ≤ 9 points for 12 consecutive weeks, and any PSQI score > 9 points after that until the end point was considered a relapse. Recurrence: any PSQI > 9 points in the consecutive 12 weeks from the onset time was considered a recurrence.

Exclusion criteria: (1) secondary or comorbid insomnia caused by various mental illnesses; (2) insomnia caused by psychoactive substances or physical diseases; (3) pregnant or lactating women.

### Study design

The general information and baseline Pittsburgh Sleep Quality Index (PSQI) were completed through the mobile phone, as well as the PSQI, Patient Health Questionnaire (PHQ-9), and Generalized Anxiety Disorder 7-item (GAD-7) scale, which were evaluated monthly thereafter using self-evaluation questionnaires. Participants completed the questionnaires and received online CBT treatment through the "Good Sleep 365" software. The content of online CBT includes sleep restriction, stimulus control, cognitive reconstruction, sleep health education, and relaxation training, among others. In addition, this APP provides scientific knowledge about insomnia, such as the harm of insomnia, insomnia treatment, and how to prevent insomnia recurrence. These pieces of information are presented to patients in the form of videos or audios for them to watch. The platform will provide new information, and patients can also review previous content.

The Good Sleep 365 platform pushes CBT-i methods and skills including sleep restriction, stimulus control, cognitive restructuring, sleep hygiene education, relaxation training, etc., and pushes an episode of content in each section every day. The content is presented to the patient in the form of video or audio, and after the patient completes the daily sleep diary, the platform provides new records (including a total of 62 videos, 4 audios, providing relaxation training guidance); Patients can also repeat the previous content, but cannot select a new audio or video, and the background will push it according to the patient's assessment results. Each episode of video or audio is about 1–4 min long.

### Evaluation tools

The general information questionnaire was a self-designed questionnaire that included basic information such as gender, age, course, and education level.

The Pittsburgh Sleep Quality Index (PSQI) was used to evaluate the patient's sleep quality over the past month. There are 19 items that constitute 7 factors, with each factor scored on a grade of 0–3, and the total score is 21 points. It can be used to classify insomnia, with 0–4 points indicating no insomnia; 5–9 points indicating mild insomnia; 10–14 points indicating moderate insomnia; and 15–21 points indicating severe insomnia^13^.

The Patient Health Questionnaire-9 (PHQ-9)^14^ was used to assess the patient's depressive symptoms. It contains 9 items with scores ranging from 0 to 3 points for each item, with a total score of 21 points. The higher the score, the more severe the depressive symptoms, with 0–4 points indicating no depressive symptoms; 5–9 points indicating mild depression; 10–14 points indicating moderate depression; and ≥ 15 points indicating severe depression.

The Generalized Anxiety Disorder 7-item (GAD-7) scale^15^ was used to screen for anxiety symptoms and assess their severity. It consists of 7 items, with each item scored on a grade of 0–3, and the total score is 21 points. 0–4 points indicate no anxiety, 5–9 points indicate mild anxiety, 10–14 points indicate moderate anxiety, and ≥ 15 points indicate severe anxiety.

### Statistical analysis

The SPSS 21.0 statistical software was used for data analysis. The demographic data of the three groups of patients were expressed as mean ± standard deviation for data that met the normal distribution, and ANOVA was used for intergroup comparison. For data that did not meet the normal distribution, the median (interquartile range) was used for statistical description, and non-parametric tests were used for intergroup comparison. The count data such as gender, education level, and course were expressed as the number of cases and percentages, and chi-square test was used. Multivariate logistic regression analysis was performed to determine the potential risk factors affecting the treatment prognosis of insomnia, and risk factors were expressed as odds ratios (ORs) and 95% confidence intervals (CIs). The level of significance was set at α = 0.05 (two-sided), and P ≤ 0.05 was considered statistically significant. *P* ≤ 0.05 is statistically significant for the difference.

### Ethical approval

This retrospective study was approved by the Ethics Committee of Seventh People's Hospital of Hangzhou (approval number: 2022-032). All participants provided written informed consent. Additionally, appropriate permissions were obtained for the usage of all instruments utilized in the research.

### Consent to participate

All subjects provided written informed consent regarding this study.

## Results

### General characteristics

**Figure 1:**
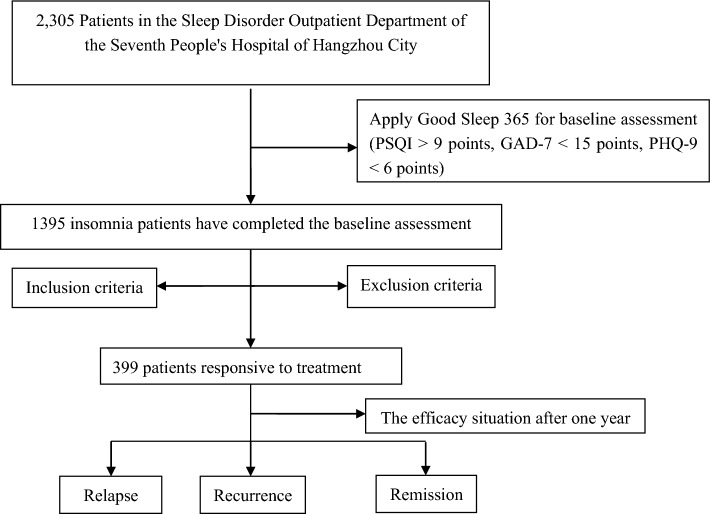
STROBE flowchart describing patients' selection. *STROBE* Strengthening the Reporting of Observational Studies in Epidemiology.

A total of 339 patients were included in the current study after the inclusion and exclusion criteria. (Fig. 1) Based on the treatment status of the patients within 1 year, they were divided into the following three groups: remission group (160 cases), relapse group (100 cases), and recurrence group (79 cases). There was no statistical difference in gender (P = 0.978), educational level (P = 0.468), and course of disease (P = 0.580) among the three groups. However, there were significant differences in age (P = 0.006) and onset time (P < 0.001) among the three groups. The remission group had a higher average age than the recurrence group and the relapse group. The onset time of the remission group was delayed compared to the other two groups (Table 1).

**Table 1:** Demographic characteristics of participants.

Characteristic	Relapse *N *(%) 79	Recurrence *N *(%) 100	Remission *N *(%) 160	*P/Z*-value
Sex				
Male	18 (22.8)	22 (22)	37 (23.1)	0.978
Female	61 (77.2)	78 (78)	123 (76.9)	
Age, year	45.52 ± 8.82	48.90 ± 11.36	50.27 ± 11.16	0.006*
≤ 30	1 (1.3)	5 (5)	10 (6.3)	
31–40	17 (21.5)	20 (20)	21 (13.1)	
41–50	32 (40.5)	28 (28)	50 (31.2)	
51–60	21 (26.6)	32 (32)	52 (32.5)	
> 60	8 (10.1)	15 (15)	27 (16.9)	
Education level				0.097
Primary school	18 (22.8)	15 (15)	22 (13.8)	
Junior high school	26 (32.9)	21 (21)	32 (20)	
Senior high school	10 (12.7)	27 (27)	38 (23.8)	
College	20 (25.3)	32 (32)	57 (35.5)	
≥ Postgraduate	5 (6.3)	5 (5)	11 (6.9)	
Duration of disorder				0.580
< 3 months	15 (18.9)	23 (23)	37 (23.1)	
3 months–1 year	19 (24.1)	17 (17)	29 (18.1)	
1–3 years	17 (21.5)	19 (19)	20 (12.5)	
3–5 years	10 (12.7)	13 (13)	23 (14.4)	
> 5 years	18 (22.8)	28 (28)	51 (31.9)	
Onset time, week^#^	8 (13)	8 (9)	13 (23)	0.000*

*Represents P < 0.05.

^#^Median (interquartile range).

### Comparison of baseline data

The GAD-7 scores of the recurrence group, relapse group, and remission group were 5.82 ± 2.36, 4.31 ± 3.37, and 4.57 ± 3.17, respectively. There was a statistical difference in the baseline GAD-7 scores among the three groups (P < 0.001). The GAD-7 score of the recurrence group was higher than that of the other two groups (Table 2).

**Table 2:** Scores of the three groups at baseline [mean ± standard deviation (SD)].

Scale	Relapse	Recurrence	Remission	*F*	*P* value
PSQI (insomnia)	14.11 ± 2.54	14.20 ± 2.62	14.31 ± 2.91	0.149	0.862
GAD-7 (anxiety)	5.82 ± 2.36	4.31 ± 3.37	4.57 ± 3.17	8.540	< 0.001*
PHQ-9 (depression)	2.61 ± 1.70	2.55 ± 1.73	2.44 ± 1.67	0.280	0.756

*Represents P < 0.05.

### Study of prognostic factors

Multivariate logistic regression analysis showed that age, high GAD-7 scores, delayed onset time, and low educational level were related to treatment prognosis (Table 3). For every 1 year increase in age, the probability of a patient's treatment effect changing from recurrence to relapse or from relapse to recurrence is higher, and the stability of the patient's therapeutic effect increases by 1.031 times. For every one-point increase in the GAD-7 score, the probability of the patient's therapeutic effect being unstable increases by 87.7%. The probability of remission for patients with only primary school education or below is 0.345 times that of those with graduate education. For every 1-week increase in onset time, the stability of the disease increases by 10.48%, which means that the longer the onset time, the more stable the disease is relative to the treatment.

**Table 3:** Risk factors for affecting treatment prognosis to insomnia according to multivariate logistic regression analysis.

Variable	Adjusted OR (95%)	*P* value
Sex		
Male	3.94 (0.56–1.56)	0.80
Female	1 [Reference]	NA
Age	1.03 (1.01–1.054)	0.001
Education level		
Primary school	0.35 (0.13–0.96)	0.41
Junior high school	0.39 (0.14–1.01)	0.05
Senior high school	0.72 (0.27–1.93)	0.52
College	1.01 (0.40–2.54)	0.99
≥ Postgraduate	1 [Reference]	NA
Duration of disorder		
< 3 months	1.27 (0.68–2.37)	0.45
3 months–1 year	0.74 (0.39–1.39)	0.35
1–3 years	0.62 (0.32–1.21)	0.16
3–5 years	0.99 (0.49–2.01)	0.98
> 5 years	1 [Reference]	NA
Onset time	1.048 (1.03–1.07)	*P* < 0.001
PSQI (insomnia)	1.01 (1.01–1.05)	0.84
GAD-7 (anxiety)	0.88 (0.82–094)	*P* < 0.001
PHQ-9 (depression)	1.06 (1.03–1.07)	0.41

*PSQI* 19-item Pittsburgh sleep quality index, *GAD-7* 7-item generalized anxiety disorder assessment, *PHQ-9* 9-item patient health questionnaire, *NA* not applicable.

## Discussion

Cognitive-behavioral therapy for insomnia (CBT-I) can effectively improve the overall sleep quality of general patients, and its efficacy lasts for at least 6 months or even longer^16,17^ However, there is little information on its long-term effects. Previous studies have shown that insomnia is a persistent disease that follows multiple trajectories over time^18,19^, and the course fluctuates over time and is related to life events, psychological factors, or health status^20^. Our results also confirmed that although it has a high remission rate during treatment, almost half of the patients (52.8%) showed fluctuations in symptoms within 1 year. These indicated that the method of using pharmacotherapy combined with mobile CBT-I to treat insomnia will also follow the natural course of insomnia over time, and fluctuations in symptoms after treatment are a common trajectory.

The prognosis of insomnia is affected by multiple factors, but our focus is to determine which individuals with specific traits will experience recurrence, relapse, and remission. The demographic data and baseline scale assessments show that there are significant differences in age and onset time among the three groups of patients. The age and onset time of the remission group were significantly higher than those of the other two groups, and the GAD-7 score of the recurrence group was higher than that of the other two groups. The multivariate logistic regression analysis showed that age and onset time were related to treatment prognosis. Specifically, for every 1-year increase in age, the probability of the patient's treatment outcome changing from recurrence to relapse or from relapse to remission increased, and the stability of the patient's treatment efficacy increased by 1.031 times, which means that the prognosis of the patient's treatment is better. For every 1-week delay in onset time, the stability of the patient's condition increased by 10.48%. In other words, the longer the onset time, the more stable the patient's condition. Therefore, being older and having a delayed onset time are protective factors for the treatment prognosis of insomnia.

Our study reveals an intriguing finding that older age serves as a protective factor for treatment prognosis. This contrasts with previous research that has often identified age as a negative predictor of treatment outcomes in sleep disorders. One possible reason for this result is that older patients tend to have a greater sense of trust in doctors and are more likely to persist with their treatment. This trust and compliance could contribute to improved treatment outcomes. Furthermore, our study suggests that delayed onset of symptoms may play a significant role. Patients with a delayed onset of insomnia often experience more pain and dissatisfaction with previous treatment attempts^21^. Individuals with early onset of treatment may prematurely stop treatment on their own, leading to a recurrence of symptoms. In addition, onset delay can potentially filter out patients who do not adhere to treatment and follow-up. Patients who adhere to treatment and follow-up typically exhibit good compliance and have a higher level of trust in their doctors. Therefore, they are more likely to cherish the treatment results once it proves effective, which positively promotes their treatment compliance and reduces the likelihood of symptom fluctuations.

The multivariate logistic regression analysis of this study showed that a higher degree of anxiety at baseline is a risk factor for treatment prognosis. Specifically, for every one-point increase in the GAD-7 score, the probability of the patient's treatment efficacy instability increased by 87.7%. This finding aligns with the current pathophysiological model, which suggests that insomnia disorder arises from the interplay between cognitive-behavioral and neurobiological factors^22^. Excessive arousal serves as the core feature of insomnia. At the cognitive level, anxiety emotions can lead to the appearance of insomnia symptoms. Excessive concern about sleep problems and attention bias to sleep-related stimuli can lead to prolonged time spent in bed, thereby perpetuating insomnia symptoms. At the neurobiological level, stressful events can impact the secretion of neurotransmitters, such as cortisol and appetite peptide, as well as alter the activity of neurons in brain regions involved in arousal promotion and sleep promotion. This disruption can result in acute cortical excessive arousal, disturbance of sleep–wake rhythm and circadian rhythm, ultimately leading to a complete alteration of the sleep–wake system. Consequently, long-term cortical excessive arousal occurs, progressing insomnia from acute to a sub-chronic and chronic states. Moreover, our study found that only individuals with a primary school or lower educational level were found to be associated with treatment prognosis which is consistent with previous studies. The probability of non-relapse in patients with a primary school or lower educational level was only about one-third of that in graduate students. This association may be attributed to the fact that individuals with higher educational levels are generally better equipped to cope with negative life events and avoid harmful behaviors^23^.

In addition, by clarifying the factors that influence the prognosis of treatment with CBT apps for insomnia, several benefits can arise for future insomnia treatment using CBT apps. CBT application is not limited to time and space, can cover more people, so that patients can receive CBT-I treatment at anytime and anywhere, through the analysis of the factors affecting the prognosis of online CBT treatment for insomnia, in clinical work for patients with different characteristics, can provide certain guidance for treatment, such as for patients with slow onset of effect, patients can be encouraged to wait for the treatment effect and the probability of subsequent recurrence and recurrence is relatively low. Understanding the factors that affect treatment prognosis allows for a more personalized approach to insomnia treatment. Healthcare professionals can tailor CBT app interventions to meet the specific needs of patients. This personalized approach can enhance treatment effectiveness and improve long-term outcomes. With knowledge of the factors that influence prognosis, healthcare providers can focus on addressing specific risk factors or implementing preventive measures. By targeting the underlying factors contributing to poor prognosis, CBT apps can be designed to provide comprehensive treatment and support. Furthermore, future CBT apps can incorporate features that support long-term follow-up and monitoring. This can include regular assessments and feedback loops to track symptom fluctuations and provide timely interventions when needed. By addressing the natural course of insomnia, CBT apps can help individuals maintain treatment gains and prevent relapse or recurrence.

This study has several limitations. Firstly, there are many factors that can affect the prognosis of insomnia. To gain a better understanding of the factors related to the development and persistence of insomnia prognosis, repeated and longer-term follow-up examinations are needed. Secondly, the different prognostic outcomes (recurrence, relapse, remission) are based only on data from subjects who completed all follow-up evaluations. Consequently, data from subjects who did not adhere to follow-up cannot be analyzed. Thirdly, the platform can only manage the training frequency of patients and cannot enforce offline completion by patients. In addition, the data used in this study are limited to individuals from Zhejiang Province, and therefore, the results cannot be representative of other regions. Future research should consider obtaining samples from a wider range of geographical areas to enhance generalizability. Fourth, without a control group, we cannot establish a cause-effect relationship and determine the specific impact of online CBT treatment on sleep disorders compared to other treatment methods. The results may indeed be applicable to general treatment predictors for sleep disorder patients rather than being specific to online CBT. Lastly, the specific factors related to the app itself were not examined in our research. Future researches are encouraged to investigate the factors specifically associated with CBT apps.

## Conclusion

Our study found that older age, primary school education or below, delayed onset time, and higher GAD scores can predict the treatment prognosis of insomnia patients. Healthcare professionals can pay special attention to these identified factors when evaluating and designing personalized treatment plans. Further researches and clinical trials are necessary to validate these findings and explore personalized treatment options for different patient subgroups.

## Data Availability

Due to restrictions related to participant confidentiality, the data are not publicly available. The data that support the findings of this study are available from the corresponding author upon reasonable request.
