# Author Correction: Mobile cognitive behavioral therapy for insomnia: analysis of factors affecting treatment prognosis

**DOI:** 10.1038/s41598-024-58107-6

**Published:** 2024-04-03

**Authors:** Jia Wei, You Xu, Hongjing Mao

**Affiliations:** Affiliated Mental Health Center and Hangzhou Seventh People’s Hospital, Zhejiang University, Hangzhou, China

Correction to: *Scientific Reports* 10.1038/s41598-024-53119-8, published online 07 February 2024

In the original version of this Article, Jia Wei, You Xu and Hongjing Mao were incorrectly affiliated with ‘Psychiatric Mental Health Center, Zhejiang University School of Medicine, Seventh People’s Hospital of Hang Zhou, Hangzhou, China’. The correct Affiliation is listed below:

‘Affiliated Mental Health Center & Hangzhou Seventh People's Hospital, Zhejiang University, Hangzhou, China.’

The original Article has been corrected.

